# Myocyte-specific enhancer factor 2C: a novel target gene of miR-214-3p in suppressing angiotensin II-induced cardiomyocyte hypertrophy

**DOI:** 10.1038/srep36146

**Published:** 2016-10-31

**Authors:** Chun-Mei Tang, Fang-zhou Liu, Jie-Ning Zhu, Yong-Heng Fu, Qiu-Xiong Lin, Chun-Yu Deng, Zhi-Qin Hu, Hui Yang, Xi-Long Zheng, Jian-Ding Cheng, Shu-Lin Wu, Zhi-Xin Shan

**Affiliations:** 1 Guangdong Cardiovascular Institute, Guangdong Provincial Key Laboratory of Clinical Pharmacology, Guangzhou 510080, China; 2 Guangdong General Hospital, Guangdong Academy of Medical Sciences, Guangzhou 510080, China; 3 Southern Medical University, Guangzhou 510515, China; 4 The Libin Cardiovascular Institute of Alberta, Department of Biochemistry & Molecular Biology, The University of Calgary, Calgary, T2N 1N4, Alberta, Canada; 5 Department of Forensic Pathology, Zhongshan School of Medicine, Sun Yat-sen University, Guangzhou 510080, China

## Abstract

The role of microRNA-214-3p (miR-214-3p) in cardiac hypertrophy was not well illustrated. The present study aimed to investigate the expression and potential target of miR-214-3p in angiotensin II (Ang-II)-induced mouse cardiac hypertrophy. In mice with either Ang-II infusion or transverse aortic constriction (TAC) model, miR-214-3p expression was markedly decreased in the hypertrophic myocardium. Down-regulation of miR-214-3p was observed in the myocardium of patients with cardiac hypertrophy. Expression of miR-214-3p was upregulated in Ang-II-induced hypertrophic neonatal mouse ventricular cardiomyocytes. Cardiac hypertrophy was attenuated in Ang-II-infused mice by tail vein injection of miR-214-3p. Moreover, miR-214-3p inhibited the expression of atrial natriuretic peptide (ANP) and β-myosin heavy chain (MHC) in Ang-II-treated mouse cardiomyocytes *in vitro*. Myocyte-specific enhancer factor 2C (MEF2C), which was increased in Ang-II-induced hypertrophic mouse myocardium and cardiomyocytes, was identified as a target gene of miR-214-3p. Functionally, miR-214-3p mimic, consistent with MEF2C siRNA, inhibited cell size increase and protein expression of ANP and β-MHC in Ang-II-treated mouse cardiomyocytes. The NF-κB signal pathway was verified to mediate Ang-II-induced miR-214-3p expression in cardiomyocytes. Taken together, our results revealed that MEF2C is a novel target of miR-214-3p, and attenuation of miR-214-3p expression may contribute to MEF2Cexpressionin cardiac hypertrophy.

Many factors including mechanical stimulus, hormones, cytokines, growth factors and pressure overload contribute to cardiac hypertrophic growth. It is well known that the pathological cardiac hypertrophy leads to cardiomyopathy and heart failure^1^. MicroRNAs, endogenous 20–23-nucleotide noncoding RNAs, are dysregulated and play important roles in cardiac hypertrophy^2^. MicroRNA-208a and microRNA-195, for example, were up-regulated in cardiac hypertrophy and sufficient to drive pathological cardiac growth when over-expressed in transgenic mice individually^3^,^4^. By contrast, microRNA-1, −150 and −181b were downregulated in cardiac hypertrophy, and their *in vitro* overexpression resulted in a decrease in the size of cardiomyocytes^4^,^5^,^6^. It was previously reported that microRNA-214 (miR-214) was up-regulated in abdominal aortic constriction (AAC)-induced cardiac hypertrophy in rats^7^,^8^, but the exact role of miR-214 in cardiac hypertrophy has not been well understood.

Myocyte-specific enhancer factor 2 (MEF2) has been implicated as a signal-responsive mediator of the cardiac transcriptional program. MEF2-binding A/T-rich DNA sequences have been identified within the promoter regions of a number of cardiac genes, such as α-MHC, myosin light chain (MLC)2v, skeletal α-actin, cardiactroponin T, -C, and -I^9^,^10^. MEF2C is upregulated during cardiac hypertrophy and is required for normal post-natal growth of the myocardium^11^. And MEF2C was reported as a target of miR-373 in glucose-induced cardiomyocyte hypertrophy^12^.

In this study, we observed a significant attenuation of miR-214-3pexpression in mouse and human hypertrophic myocardium. Enforced enhancement of miR-214-3p ameliorated angiotensin II (Ang-II) infusion-induced cardiac hypertrophy in mice. Our results demonstrated that mouse miR-214-3p negatively regulated MEF2C expression by directly targeting the 3′untranslated region (UTR) of MEF2C mRNA. Either miR-214-3p mimic or MEF2C siRNA could efficiently inhibit Ang-II-induced hypertrophy in mouse cardiomyocytes. Furthermore, we demonstrated a role for the NF-κB pathway in the upregulation of miR-214-3p in hypertrophic cardiomyocytes induced by Ang-II. Our data suggest that MEF2C is a novel target of miR-214-3p in myocardial hypertrophy, and enhancement of miR-214-3p expression may be protective against myocardial hypertrophy.

## Methods

### Ethics Statement

Male C57BL/6 mice weighing 20 ± 3 g and 1- to 3-d old newborn C57BL/6 mice (License number SCXK (YUE) 2004–0011, Department of Experimental Animal Research Center, Sun Yat-sen University, Guangzhou, China) were used in the current studies. Mice were housed under a 12-h light/dark cycle under pathogen-free conditions and with free access to standard mouse chow and tap water. This study conformed to the Guide for the Care and Use of Laboratory Animals published by the US National Institutes of Health (8th Edition, National Research Council, 2011). All methods and experimental protocols in the present program were also approved by the research ethics committee of Guangdong General Hospital (the approval number: No. GDREC2010093A).

The human ventricular samples were stored and donated by the tissue bank of the Department of Forensic at Sun Yat-sen University in Guangzhou, China.

### Animal studies

According to previously described methods, we established mouse cardiac hypertrophy models of Ang-II (1.46 mg/kg/d, 14 d) infusion^13^ and pressure-overloading by transverse aortic constriction (TAC)^14^. Mice were anesthetized through the intraperitoneal application of sodium pentobarbital (50 mg/kg), followed by implantation of the Ang-II mini-osmotic pump (alzet model 2002, Cupertino, CA, USA) or the TAC surgery. The adequacy of anesthesia was confirmed by the absence of reflex response to foot squeeze. Body temperature was maintained at 37 ± 0.5 °C during surgery. At the end of the experiments, mice were killed with the intraperitoneal injection of an overdose of sodium pentobarbital (200 mg/kg).

To investigate the effect of miR-214-3p on Ang-II-induced hypertrophy *in vivo*, 24 C57BL/6 mice were randomized into 4 groups: (1) agomiR-negative control (NC) + saline, (2) agomiR-NC + Ang-II (NC agomir with Ang-II infusion), (3) agomiR-214 + saline (miR-214-3p agomir with saline infusion), and (4) agomiR-214 + Ang-II (miR-214-3p agomir with Ang-II infusion). All agomirs were purchased from (RiboBio, Guangzhou,China).The amount of 20 nmol NC agomir or miR-214-3p agomir was delivered into each mouse via tail vein injection at 4 interval time points within 14 d.

### Echocardiography

Left ventricular (LV) function variables were assessed by transthoracic echocardiography 2 weeks after the mini-osmotic pump implant surgery. After the induction of light general anesthesia, the mice received transthoracic two-dimensional (2D) guided M-mode echocardiography (Technos, MP, 8.5-MHz transducer). From the cardiac short axis (papillary level), the end-systolic and end-diastolic LV posterior wall diameters (LVPWs and LVPWd), the end-systolic and end-diastolic LV internal diameters (LVIDs and LVIDd), ejection fraction (EF), and fractional shortening (FS) were measured. Echocardiographic measurements were averaged from at least three separate cardiac cycles.

### Wheat germ agglutinin (WGA) staining

Mice were sacrificed with an overdose of sodium pentobarbital (200 mg/kg, ip) at the end of experiments. The mouse heart was excised, and the LV myocardium was fixed overnight in 10% formalin.

In this study, mouse or human ventricular samples were embedded in paraffin and cut into 4 μm-thick sections. Tissue sections were mounted on the regular glass slides and stained with 1.0 mg/mL Alexa Fluor^®^ 488 conjugate of WGA solution (MolecularProbes, Eugene, Oregon, USA) to demonstrate the size of cardiomyocytes in mouse or human ventricular myocardium.

### Primary culture of mouse ventricular cardiomyocytes and treatments

Neonatal mouse ventricular cells (NMVCs) were isolated from the hearts of 1–3-d-old newborn C57BL6 mice as described previously^15^. The newborn mice were killed by cervical dislocation. Isolated NMVCs were plated onto 12-well plates and maintained for 48 h in DMEM/F-12 supplemented with 10% FBS (Gibco, New York, NY). NMVCs were incubated with 10^−8^ M Ang-II for 48 h to induce the hypertrophic phenotype. Cells were treated with NF-κB inhibitor JSH23 (5 μM) or QNZ (5 nM). Cells were transfected with 50 nM scramble ormiR-214-3p mimic, or 50 nMsiRNA for MEF2C or NF-κB P65 (Ribobio, Guangzhou, China) by oligofectamine reagent (Invitrogen, Carlsbad,CA). As indicated, NMVCs were infected with the following recombinant adenovirus, respectively: rAd-GFP, rAd-IKK-β and Ad-IKB-α adenovirus (MOI 5).

### FITC-phalloidin staining

Cultured NMVCs were fixed in 3.7% formaldehyde and permeabilized in 0.1% Triton X-100 for 10 min, respectively, followed by incubation with blocking solution for 40 min and subsequently with FITC-phalloidin (10 μg/ml, Sigma-Aldrich) at 37 °C. Confocal micrographs were obtained using a Leica SP5 confocal microscope (Leica, Mannheim, Germany). Cell size (total area) was quantified using MiVnt imaging software (Weiyu, Zhuhai, China).

### Quantitative miRNA and mRNA measurements

Reverse-transcription quantitative PCR (RT-qPCR) for miR-214-3p was performed on cDNA generated from 0.5 μg total RNA according to the manufacturer’s protocol (Ribobio, China). For the detection of mRNA expression of coding genes, the first-strand cDNAs were generated from 1.5 μg total RNA using a mixture of oligo (dT)_15_ and random primers with superscriptreverse transcriptase (Invitrogen, Carlsbad, CA). To normalize RNA content, U6 was used for miR-214-3p template normalization and GAPDH was used for coding genes template normalization. PCR was performed with the ViiA7 Quantitative PCR System (Applied Biosystems, Carlsbad, CA). The 2^−ΔΔCt^ method was used to calculate relative expression levels of miR-214-3p and coding genes^16^. PCR primers for miR-214-3p, U6 and coding genes are shown in Supplementary Table 1.

### Western blot analysis

The amount of 40 μg protein prepared from mouse myocardium or NMVCs was used in a standard western blot analysis. The polyvinylidene fluoride (PVDF) membrane binding sample protein was incubated with a high affinity anti-ANP antibody (1:500 dilution), anti-β-MHC antibody (1:1000), anti-MEF2C antibody (1:1000)(Abcam, Cambridge, MA), anti-p-NF-κB P65 (1:1000), anti-NF-κB P65 (1:1000)(Cell SignalingTechnology, Beverly, MA, USA), respectively. An anti-GAPDH antibody (1:2000) (Santa CruzBiotechnology, Santa Cruz, CA) was used to detect the level of GAPDH as an internal control. Protein was visualized using the ECL Plus detection system (GE Healthcare, Waukesha, WI).

### Dual luciferase assay for MEF2C target identification

According to our previous report^17^, the recombinant luciferase reporter plasmid containing the potential miR-214-3p binding site sequence of MEF2C gene was constructed. Using a site-directed mutagenesis kit (TransGen, Beijing, China), the miR-214-3p binding site sequence GTCGTCC was replaced with GAGCACC to construct a recombinant luciferase reporter plasmid containing the mutant potential miR-214 binding sequence. Human embryonic kidney (HEK) 293 cells (3 × 10^5^ cells per well in the 12-well plate) were cotransfected with 200 ng of recombinant luciferase reporter plasmid, 50 nM miR-214-3p mimic, and 20 ng of pRL-TK plasmid as an internal control (Promega, Madison, WI). Activities of firefly luciferase (FL) and Renilla luciferase (RL) were measured 24 h after transfection. The relative ratio of the FL/RL was used to indicate the suppression of MEF2C by miR-214-3p.

### Statistical analysis

The data are presented as the means ± s.e.m. In each experiment, all determinations were performed at least in triplicate. Statistical significance between two measurements was determined by the two tailed unpaired Student’s t test, and among groups, it was determined by one-way ANOVA. A value of *p* < 0.05 indicated the significant difference.

## Results

### Decreased expression of miR-214-3p in the hypertrophic myocardium and cardiomyocyte

**Figure 1:**
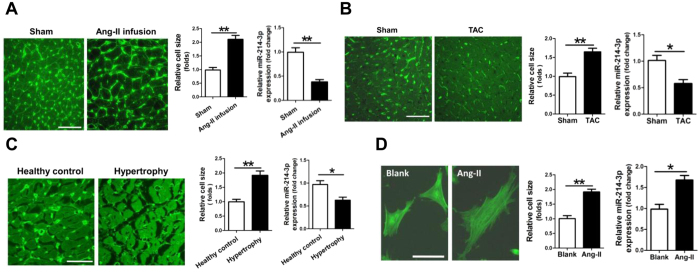
MicroRNA-214-3p (miR-214-3p) expression in the hypertrophic myocardium and cardiomyocytes. WGA staining assay of cardiomyocytes in the hypertrophic myocardium of a mouse model of Ang-II-infusion-induced hypertrophy (**A**) and TAC-induced hypertrophy (**B**), and the patients with hypertrophy (**C**). FITC-phalloidin staining of Ang-II-induced hypertrophic NMVCs (**D**). Expression of miR-214-3p in mouse myocardium and cardiomyocytes by RT-qPCR assay. The scale bar was 50 μm. Data are shown as mean ± sem, *p <  0.05, **p <  0.01. N = 5–8.

An animal model of hypertrophy was established in mice with Ang-II infusion or transverse aortic constriction (TAC), respectively. WGA staining results revealed that the cell size of cardiomyocytes was significantly increased in the myocardium of the mouse Ang-II infusion model or TAC model (Fig. 1A,B). The cell size of cardiomyocytes was also markedly increased in the myocardium of patients with hypertrophy (Fig. 1C). Consistently, our results showed that the expression of miR-214-3p was decreased in the above mouse and human hypertrophic myocardium (Fig. 1A–C). The FITC-Phalloidin staining showed that a cell model of Ang-II-induced hypertrophy was established in NMVCs. In this *in vitro* model, we found thatmiR-214-3p expression was upregulated in Ang-II-induced hypertrophic mouse cardiomyocytes (Fig. 1D).

### MiR-214-3p attenuates Ang-II-induced hypertrophic phenotype *in vivo* and *in vitro*

**Figure 2:**
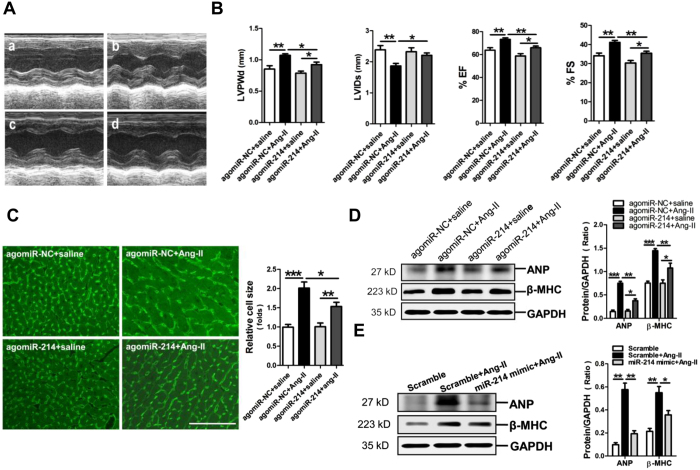
Overexpression of microRNA-214-3p (miR-214-3p) attenuates Ang-II-induced cardiac hypertrophy *in vivo* and *in vitro*. (**A**) Representative echocardiographs of mouse hearts. (a) agomiR-NC + saline, (b) agomiR-NC + Ang-II, (c) agomiR-214 + saline, (d) agomiR-214 + Ang-II. (**B**) The representative variables of echocardiograph assay in mice, including LVPWd, LVIDs, EF and FS. (**C**) WGA staining assay of cardiomyocytes in mouse myocardium. Protein expression of ANP and β-MHC in mouse myocardium (**D**) and NMVCs (**E**) by western blot assay. The scale bar was 50 μm. Data are shown as mean ± sem, *p < 0.05, **p < 0.01, ***p < 0.001. N = 6 in (**A–D**), N = 3 in (**E**).

To further establish the role of miR-214 downregulation in Ang-II-induced cardiac hypertrophic myocardium, we determined if overexpression of miR-214-3p via tail vein injection of miR-214-3p agomir had protective effects on the cardiac hypertrophy. As expected, the level of miR-214-3p was significantly increased in the myocardium of mice received injection of miR-214-3p agomir (Supplimentary Fig. 1). Echocardiography was performed to reveal cardiac structure and function changes in Ang-II infusion mice with or without miR-214-3p agomir injection (Fig. 2A). The thickened LV walls (LVPWd, LVPWs) and decreases in the LV internal diameters (LVIDd, LVIDs) were observed in Ang-II infusion mice, but miR-214-3p agomir injection efficiently reversed the increase of LVPW and the decrease of LVID in Ang-II infusion mice (Fig. 2B, Supplimentary Fig. 1). In addition, the compensatory increases of ejection fraction (EF) and fractional shortening (FS) were markedly relieved by miR-214-3p agomir delivery in Ang-II infusion mice (Fig. 2B).

The WGA staining results showed that cell size of cardiomyocyte in the myocardium was markedly increased in Ang-II infusion mice, which was reversed by enforced expression of miR-214-3p (Fig. 2C). Meanwhile, our western blot results demonstrated that ANP and β-MHC protein expression in mouse myocardium in response to Ang-II infusion was also suppressed by miR-214-3p injection (Fig. 2D). Consistently, miR-214-3p also markedly attenuated Ang-II-induced ANP and β-MHC protein expressions in NMVCs (Fig. 2E).

### Verification of MEF2C as a target gene of miR-214-3p

**Figure 3:**
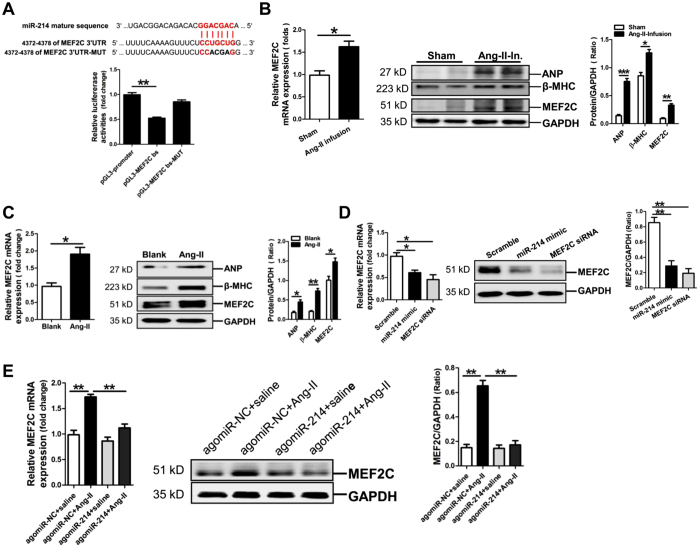
MicroRNA-214-3p (miR-214-3p) negatively modulates MEF2C expression. (**A**) Verification of MEF2C as a target gene of miR-214-3p by the dual luciferase reporter system. Predicted miR-214-3p seed matches to the sequence in the 3′-UTR of MEF2C gene mRNA. The seed sequence of miR-214-3p is UGGAAUG, and the complementary nucleotide sequences are shown in red words. Data are shown as mean ± sem, **p < 0.01 vs pGl3-promoter vector control, N = 3. (**B**) MEF2C mRNA and protein expression in the myocardium of Ang-II-infusion mouse model of cardiac hypertrophy were assessed by RT-qPCR assay and western blot assay, respectively. Data are shown as mean ± sem, *p < 0.05, **p < 0.01, ***p < 0.001. N = 5-8. MEF2C mRNA and protein expression in Ang-II-induced mouse cardiomyocytes (**C**) and mouse cardiomyocytes transfected with miR-214-3p mimic or MEF2C siRNA (**D**). Data are shown as mean ± sem, *p < 0.05, **p < 0.01. N = 3. (**E**) MEF2C mRNA and protein expression in the myocardium of Ang-II-infusion mouse model of cardiac hypertrophy with overexpression of miR-214-3p. Data are shown as mean ± sem, **p < 0.01. N = 6.

Analysis of the databases Mirdb (www.mirdb.org) and TargetScan-Vert (www.targetscan.org) showed that MEF2C was a potential target gene of miR-214-3p. The matching position for miR-214-3p within 3′-UTR of the targeted mRNAs is shown in Fig. 3A. The dual luciferase assay demonstrated that miR-214-3p significantly reduced the luciferase activity, but not when 4372–4378 binding sequence in the 3′-UTR of MEF2C gene was mutated (Fig. 3A). Our RT-qPCR and western blot results revealed that MEF2C mRNA and protein expression was significantly increased in mouse hypertrophic myocardium and Ang-II-treated NMVCs accompanied by significant increase in ANP and β-MHC protein expression (*p* < 0.05, *p* < 0.01, respectively) (Fig. 3B,C). Next, we examined the expression of MEF2C in NMVCs transfected with miR-214-3p mimic or MEF2C siRNA. Compared with the negative scramble control, MEF2C mRNA and protein expression was significantly reduced in miR-214-3p mimic-modified NMVCs and also in NMVCs transfected with MEF2C siRNA (*p* < 0.05, *p* < 0.01, respectively) (Fig. 3D). Moreover, MEF2C mRNA and protein expression in the myocardium of Ang-II infusion mice could also be reversed by enforced increase of miR-214-3p (Fig. 3E).

### MiR-214-3p and MEF2C siRNA attenuate the hypertrophic phenotype in Ang-II-treated cardiomyocytes

**Figure 4:**
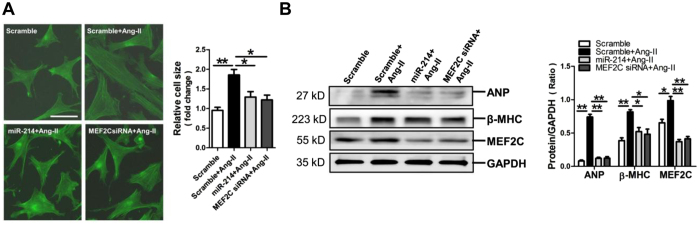
MicroRNA-214-3p (miR-214-3p) inhibits hypertrophic phenotype of NMVCs *in vitro*. (**A**) Morphologies of Ang-II-treated NMVCs as revealed by FITC-phalloidin staining. The scale bar was 50 μm. (**B**) ANP, β-MHC and MEF2C protein expression in NMVCs were assessed by western blot assay. Data are shown as mean ± sem, *p < 0.05, **p < 0.01. N = 3.

MiR-214-3p mimic and MEF2C siRNA were transfected into NMVCs, followed by FITC-phalloidin staining assay and examining the expressions of hypertrophy-related genes. FITC-phalloidin staining results demonstrated that cell size changes of Ang-II-treated NMVCs were markedly reversed by miR-214-3p mimic and MEF2C siRNA, respectively (*p* < 0.05) (Fig. 4A). Consistently, our western blot results showed that protein expression of ANP, β-MHC and MEF2C could be inhibited by miR-214-3p mimic and MEF2C siRNA in Ang-II-induced NMVCs (*p* < 0.05, *p* < 0.01, respectively) (Fig. 4B).

### MiR-214-3pis up-regulated by Ang-II through the NF-κB pathway

**Figure 5:**
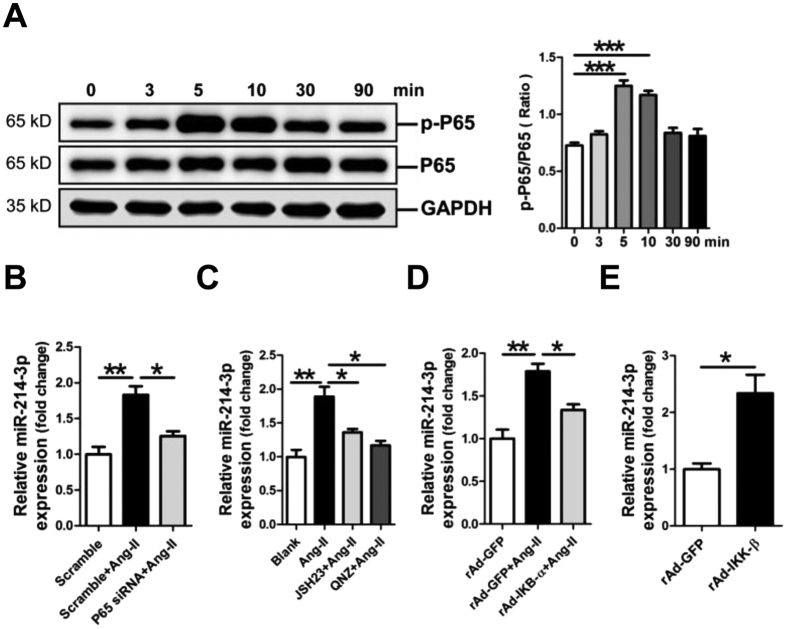
Up-regulation of microRNA-214-3p (miR-214-3p) in NMVCs through the NF-κB pathway. (**A**) Time-dependent activation of NF-κB signaling in Ang-II-treated NMVCs. MiR-214-3p expression in Ang-II-induced NMVCs with knockdown of P65 (**B**), or with pre-treatment with the NF-κB inhibitor JSH23 or QNZ, respectively (**C**), or with overexpression of IKB-α (**D**) was assessed by RT-qPCR assay. Additionally, miR-214-3p expression in NMVCs with overexpression of IKK-β was also detected (**E**). Data are shown as mean ± sem, *p < 0.05, **p < 0.01, ***p < 0.001. N = 3.

It was previously reported that NF-κB activation suppresses miR-214 transcription in hepatocellular carcinoma cells^18^. Therefore, we investigated whether modulation of miR-214-3p in hypertrophic cardiomyocytes was also mediated through the NF-κB pathway. We first examined time-dependent activation of the NF-κB pathway in Ang-II-treated NMVCs. Our western blot results showed that the phosphorylation level of NF-κB P65 was significantly increased in NMVCs at 5 and 10 min in response to Ang-II treatment (Fig. 5A). Knockdown of P65 by P65 siRNA inhibited Ang-II-promoted miR-214-3p expression in NMVCs (Fig. 5B). Next, we pre-treated NMVCs before Ang-II with NF-κB P65 inhibitor JSH23 or QNZ for 0.5 h, followed by RT-qPCR analysis. Our data demonstrated that treatment with either JSH23 or QNZ prevented Ang-II-induced miR-214-3p expression (Fig. 5C). Moreover, enforced expression of IKB-α, which inactivates the NF-κB P65 pathway, attenuated Ang-II-stimulated miR-214-3p expression in NMVCs (Fig. 5D). However, enforced expression of IKK-β, which activates the NF-κB P65 pathway, enhanced miR-214-3p expression (Fig. 5E). Collectively, our results suggest that up-regulation of miR-214-3p in Ang-II-induced hypertrophic cardiomyocytes results from activation of the NF-κB pathway.

## Discussion

It was previously reported that miR-214 was upregulated during the ischemic injury (IR), and genetic deletion of miR-214 in mice caused loss of cardiac contractility and increased apoptosis in response to IR injury. Mechanistically, the cardioprotective role of miR-214 during IR injury was attributed to its repression on sodium/calcium exchanger 1 (Ncx1) and regulation of cardiomyocyte Ca^2^^+^ homeostasis^19^. Similarly, miR-214 was upregulated in H_2_O_2_-treated cardiac myocytes, and overexpression of miR-214 alleviated H_2_O_2_-induced cardiac cell apoptosis through suppressing the phosphatase and tensin homolog deleted on chromosome 10 (PTEN)^20^. It was also reported that miR-214 was involved in cardiac hypertrophy^7^,^8^. It was observed that miR-214 was up-regulated in phenylephrine (PE)-induced hypertrophic cardiomyocytes and in a rat model of AAC-induced cardiac hypertrophy^7^,^8^. Enforced expression of miR-214 could increase cell size of rat cardiomyocytes and the expression of ANP, Acta1 and β-MHC^7^. Moreover, obvious cardiac hypertrophy phenotype was obtained in the transgenic mice with cardiomyocyte-specific overexpression of miR-214^21^. The enhancer of zeste homolog 2 (EZH2) was reported to be a direct target of miR-214 during cardiac hypertrophy *in vitro* and *in vivo*^7^,^21^. However, a recent study revealed that interaction of EZH2 and primary microRNA-208b (pri-miR-208b) regulates hypertrophic gene expression, and attenuation of EZH2 can restore antisense β-MHC and α-MHC gene expression, and inhibit cardiac hypertrophy^22^. To date, the role and potential target gene of miR-214 in cardiac hypertrophy have not been well-illustrated.

In the present study, we observed that miR-214-3p was significantly down-regulated in the hypertrophic myocardium of a mouse Ang-II infusion model and a mouse TAC model. Additionally, attenuation of miR-214-3p was also observed in the myocardium of patients with hypertrophy. We have also shown that miR-214-3p was upregulated in Ang-II-induced hypertrophic mouse cardiomyocytes. Thereafter, we delivered miR-214-3p agomir via tail vein to increase miR-214-3p level in mouse myocardium to further confirm the role of miR-214-3p downregulation in Ang-II-induced cardiac hypertrophy. As expected, the compensatory increase of heart function was restored when cardiac hypertrophy was attenuated by miR-2014-3p in a mouse Ang-II infusion model. Consistently, miR-214-3p also significantly inhibited ANP and β-MHC expression in Ang-II-treated mouse cardiomyocytes. Therefore, our data have demonstrated a protective role of miR-214-3p in cardiac hypertrophy, instead of a pro-hypertrophy effect.

Notably, the activation of the MEF2 family members (MEF2A, B, C and D) of transcription factors plays prominent roles in the regulation of cardiac hypertrophy and remodeling^23^,^24^,^25^. It was reported that the transgenic expression of negative dominants of MEF2 prevents chamber dilation and mechanical dysfunction in calcineurin-induced hypertrophy^24^. In addition, MEF2C is activated in pressure- or volume-overloaded cardiac hypertrophy^10^. It was shown that the knockdown of myocardial MEF2C attenuates the hypertrophic responses to pressure overload in mice^26^. Consistently, our present study has shown that MEF2C was significantly increased in the hypertrophic myocardium of the mouse Ang-II infusion model and Ang-II-treated mouse cardiomyocytes, and silencing of MEF2C by siRNA suppressed Ang-II-induced cardiomyocyte hypertrophy.

Our current study has provided several lines of evidence to support the notion that miR-214-3p inhibits cardiac hypertrophy through targeting MEF2C. First, the *in silico* prediction indicated that MEF2C was a potential target of miR-214-3p, and the dual luciferase assay revealed that miR-214-3p specifically bound to the 4372-4378 site in the 3′-UTR of MEF2C. Additionally, miR-214-3p mimic inhibited MEF2C expression at both mRNA and protein levels in mouse cardiomyocytes. Importantly, the upregulation of MEF2C mRNA and protein was reversed by miR-214-3p agomir injection in the myocardium in the mouse Ang-II infusion model. Moreover, in parallel with the findings with MEF2C siRNA, over-expression of miR-214-3p decreased the cell size of mouse cardiomyocytes and the expression of ANP and β-MHC and suppressed Ang-II-induced cardiomyocyte hypertrophy.

Previous reports suggested a role for the NF-κB signaling in cardiac hypertrophy^27^,^28^,^29^. Our present data have confirmed that the pathway involving NF-κB P65 was activated in Ang-II-treated mouse cardiomyocytes. We used NF-κB P65 siRNA, NF-κB P65 inhibitor JSH23 and QNZ to further verify the role of the NF-κB P65 pathway in Ang-II-promoted upregulation of miR-214-3p in mouse cardiomyocytes. Moreover, we took advantage of adenovirus-mediated overexpression of IKK-β and IKB-α in mouse cardiomyocytes, respectively. Consistently, activation of NF-κB P65 by IKK-β overexpression enhanced miR-214-3p expression in mouse cardiomyocytes. However, inactivation of NF-κB P65 by IKB-α overexpression inhibited Ang-II-promoted miR-214-3p expression. Interestingly, it was reported that miR-214 expression was negatively modulated by the NF-κB P65 pathway in hepatocellular carcinoma (HCC) cells^18^. This opposite finding may result from different types of cells. Nevertheless, the mechanism underlying the down-regulation of miR-214-3p in human and mouse hypertrophic myocardium warrants further investigation.

Taken together, our results have demonstrated that miR-214-3p is down-regulated in cardiac hypertrophy, and miR-214-3p ameliorates cardiac hypertrophic responses *in vivo* and *in vitro*. Our data have also revealed that miR-214-3p inhibits hypertrophic phenotype in cardiomyocytes through down-regulation of MEF2C expression. We also conclude that activation of the NF-κB signaling pathway contributes to the upregulation of miR-214-3p in Ang-II-induced mouse cardiomyocytes. Therefore, the present study suggests that miR-214-3p might be a potential target for prevention and treatment of cardiac hypertrophy.

## Additional Information

**How to cite this article**: Tang, C.-M. *et al*. Myocyte-specific enhancer factor 2C: a novel target gene of miR-214-3p in suppressing angiotensin II-induced cardiomyocyte hypertrophy. *Sci. Rep*. **6**, 36146; doi: 10.1038/srep36146 (2016).

**Publisher’s note:** Springer Nature remains neutral with regard to jurisdictional claims in published maps and institutional affiliations.

## Supplementary Material

Supplementary Information
