# Supplementary material for: Myocyte-specific enhancer factor 2C: a novel target gene of miR-214-3p in suppressing angiotensin II-induced cardiomyocyte hypertrophy
Source: Sci Rep. 2016 Oct 31;6:36146. doi: 10.1038/srep36146 (PMC5087095; doi:10.1038/srep36146)
Supplement: Supplementary Information [file srep36146-s1.pdf]

Myocyte-specific enhancer factor 2C: a novel target gene of miR-214-3p in suppressing angiotensin II-induced cardiomyocyte hypertrophy

Chun-Mei Tang[1,2,3]*, Fang-zhou Liu[1,2]*, Jie-Ning Zhu[1,2], Yong-Heng Fu[1,2], Qiu-Xiong Lin[1,2], Chun-Yu Deng[1,2], Zhi-Qin Hu[1,2,3], Hui Yang [1,2], Xi-Long Zheng[4], Jian-Ding Cheng[5], Shu-Lin Wu[1,2] and Zhi-Xin Shan[a,1,2]

[1] Guangdong Cardiovascular Institute, Guangdong Provincial Key Laboratory of Clinical Pharmacology, Guangzhou 510080, China

[2] Guangdong General Hospital, Guangdong Academy of Medical Sciences, Guangzhou 510080, China

[3] Southern Medical University, Guangzhou 510515, China

[4]The Libin Cardiovascular Institute of Alberta, Department of Biochemistry & Molecular Biology, The University of Calgary, Calgary, Alberta, Canada T2N 1N4

[5]Department of Forensic Pathology, Zhongshan School of Medicine, Sun Yat-sen University, Guangzhou 510080, China

[a]Email: zhixinshan@aliyun.com

[*] These authors contributed equally to this work.

Supplementary

Table 1　Primers used in qRT-PCR assay

Gene	Sequence (5′- 3′)	Product size (bp）
MEF2C	F, GGTAACACAGGCGGTCTGAT	197
	R, ATAAGAACGCGGAGATCTGG	
GAPDH	F, CAAGAAGGTGGTGAAGCAGG	200
	R, CCACCCTGTTGCTGTAGCC	
Mature miR-214-3p	RT, GTCGTATCCAGTGCGTGTCGTGGAGT CGGCAATTGCACTGGATACGACACTGCCTG	76
	F, GTCCGCACAGCAGGCACAGACAGGCAGT	
	R, GTGCGTGTCGTGGAGTC	
U6	RT, GTCGTATCCAGTGCGTGTCGTGGAGT CGGCAATTGCACTGGATACGAC	160
	F, GTCCGCGTGCTCGCTTCGGCAGC	
	R, GTGCGTGTCGTGGAGTC	

A B

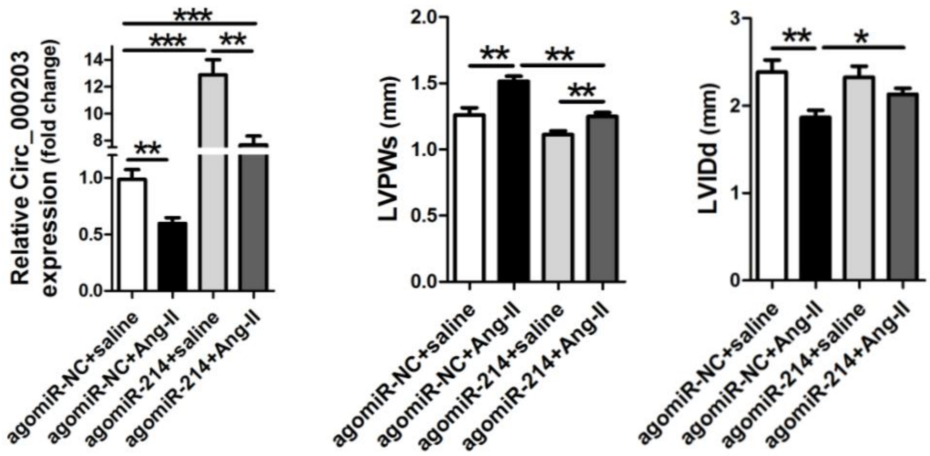

Figure 1 Determination of miR-214-3p in mouse myocardium and representative variables of echocardiograph assay in mice. A. Expression of miR-214-3p in mouse myocardium. B. The variables of echocardiograph assay, LVPWs and LVIDd, in mice. Data are shown as mean ± sem, *p<0.05, **p<0.01, ***p<0.001. N=6.
